# Blepharospasmus Caused by Excessive Efforts of Accommodation

**Published:** 1878-03

**Authors:** F. C. Hotz


*Blepharospasmus, Caused by Excessive Efforts of Accommodation.*

On November 24, 1877, Charles L., aged 12 years, was brought to my office with a pronounced blepharospasmus. He could not hold his lids still for a second; they were opening and closing in rapid alternations. These spasms of the orbicularis muscles had been lasting five days, and the boy was obliged to leave school. His father observed that the winking was less in the dark, but grew worse in bright light or by the attempt at reading. There was no external inflammation about the eyes; the media were perfectly clear; fundus normal; but the ophthalmoscope showed a degree of hypermetropia (1-18). The boy was in good health, and had not been sick before; but through hard study he had evidently overtaxed his power of accommodation and produced a state of morbid excitability of the ciliary muscle, and, as a secondary disturbance, the spasmodic action of the lid muscles.

The ciliary muscle, quieted by atropia, the blepharospasmus disappeared, so that on November 27 the eyes appeared as natural as ever. Then the sight could be tested; it was found that the boy could read test types No. XX. at 20 feet better with +18 than with the naked eye. He was directed to use glasses for reading and writing. F. C. Hotz.

---

Prof. Lister is now using and recommending horse hair for drainage. He prefers it to either rubber tubes or catgut.

